# Stress-Activity Mapping: Physiological Responses During General Duty Police Encounters

**DOI:** 10.3389/fpsyg.2019.02216

**Published:** 2019-10-04

**Authors:** Simon Baldwin, Craig Bennell, Judith P. Andersen, Tori Semple, Bryce Jenkins

**Affiliations:** ^1^Department of Psychology, Carleton University, Ottawa, ON, Canada; ^2^Royal Canadian Mounted Police, Ottawa, ON, Canada; ^3^Department of Psychology, University of Toronto Mississauga, Mississauga, ON, Canada

**Keywords:** police, occupational stress, physiological reactivity, heart rate, use-of-force

## Abstract

Policing is a highly stressful and dangerous profession that involves a complex set of environmental, psychosocial, and health risks. The current study examined autonomic stress responses experienced by 64 police officers, during general duty calls for service (CFS) and interactions with the public. Advancing previous research, this study utilized GPS and detailed operational police records as objective evidence of specific activities throughout a CFS. These data were then used to map officers’ heart rate to both the phase of a call (e.g., dispatch, enroute) and incident factors (e.g., call priority, use-of-force). Furthermore, physical movement (i.e., location and inertia) was tracked and assisted in differentiating whether cardiovascular reactivity was due to physical or psychological stress. Officer characteristics, including years of service and training profiles, were examined to conduct a preliminary exploration of whether experience and relevant operational skills training impacted cardiovascular reactivity. Study results provide foundational evidence that CFS factors, specifically the phase of the call (i.e., arrival on scene, encountering a subject) and incident factors (i.e., call priority, weapons, arrest, use-of-force), influence physiological stress responses, which may be associated with short-term performance impairments and long-term health outcomes. Implications of research findings for operational policing, police training, and health research are discussed.

## Introduction

Policing is a highly stressful and dangerous profession that involves a complex set of environmental, psychosocial, and health risks (Pinizzotto et al., 2006; Gershon et al., 2009; Chopko and Schwartz, 2012; Violanti, 2014; Andersen et al., 2016a). Against a background of less dangerous tasks, officers are required to respond to violent and life-threatening situations, often encountering novel, ambiguous, and rapidly unfolding events (Fridell and Binder, 1992). It is under these conditions that officers are required to make decisions, sometimes in a split-second, and act to protect the public and themselves (Artwohl, 2002). The current study examines physiological responses experienced by police officers, during general duty calls for service (CFS) and interactions with the public. The aim of the study is to provide novel evidence of how frequently officers experience high physiological stress responses and examine the influence of the phase of the call (e.g., dispatch, enroute) and incident factors (e.g., call priority, use-of-force) on physiological arousal. The study will also explore whether experience and relevant operational skills training impact cardiovascular reactivity.

When presented with a threatening stimulus (whether real or perceived) the body engages in a series of automatic physiological processes (LeDoux and Pine, 2016). Ledoux and Pine described two pathways to the threat, or “fear” response, more colloquially known as the “fight-or-flight” response. The pathways are: (1) behavioral and physiological stress responses and (2) fearful feelings in higher order cognitive processing. Under elevated levels of stress, the engagement of the first path, automatic physiological processing, happens within sub-cortical structures of the brain’s limbic system. The second path engages higher order cortical cognitive processing, generating conscious feelings, such as fear or other related emotions, in response to a threat (Fenici et al., 2011; LeDoux and Pine, 2016). The fight-or-flight response is implicit (i.e., below conscious awareness) and is the default human response to threat in order to maximize survival by immediately preparing the body to fight or flee without the need for higher-order cognitive processing (Thayer and Sternberg, 2006; LeDoux and Pine, 2016).

During the fight-or-flight response, two central physiological processes are engaged to mobilize the body to meet the demands of the situation and suppress unnecessary functions (e.g., reproduction, growth; McEwen, 1998; Kemeny, 2003). As described in detail by McEwen (1998) and Lovallo (2016), the sympatho-adrenal response results in a wide-spread, powerful reaction, which includes the release of neurotransmitters and hormones. The other physiological process is the engagement of the autonomic nervous system (ANS), which is made up of two branches – the sympathetic (SNS) and parasympathetic (PNS) divisions.

Perceived threats are associated with an increase in SNS activation and, typically, the suppression of the PNS, which is associated with relaxation, focused attention, and stabilization (Berntson and Cacioppo, 2004). As reviewed by Lovallo (2016), when the SNS is activated, catecholamines such as norepinephrine and epinephrine (i.e., adrenaline) are released. Simultaneously, the hypothalamic-pituitary-adrenal (HPA) axis is activated, which results in the rapid release of epinephrine and cortisol from the adrenal glands (Lovallo, 2016). Cortisol stimulates glucose production and mobilizes fatty acids to encourage higher blood sugar and prepare for energy expenditure (Anderson et al., 2002; Sharps, 2016). The surge of these catecholamines, stress hormones, and glucose through the bloodstream stimulate increased respiration, heart rate, and blood pressure (Tsigos and Chrousos, 2002; Chrousos, 2009). The increased blood flow, oxygenation, and energy are then directed in the highest concentration to the brain, heart, and large muscles (Tsigos and Chrousos, 2002). Conversely, blood flow to other areas (e.g., digestive system), which are not required to respond to a threat, are inhibited. Thus, activation of this stress system leads to an increase in strength, resistance, and attention to improve chances for survival in the short-term (Tsigos and Chrousos, 2002; Fenici et al., 2011). However, chronic, or maladaptive autonomic activation can be detrimental to health over the long-term (McEwen, 1998). The ways in which chronic stress may be detrimental to the health of police officers has been examined (Violanti et al., 2006a,b). Longitudinal studies indicate that police officers experience dysregulation in HPA axis functioning associated with occupational stressors (Violanti et al., 2017). Furthermore, police officers are more likely to be diagnosed with chronic health conditions such as heart and metabolic disease than their civilian peers (Violanti et al., 2006b). However, there is a lack of studies examining the impact of acute stress on health among police officers.

Research has suggested that SNS arousal that matches situational demands (not too high or too low) is beneficial for performing optimally during threatening situations, as it can result in heightened sensory perceptions, rapid decision-making, and improved cognitive functioning (Cahill and Alkire, 2003; Hansen et al., 2009; Jamieson et al., 2010; Lambourne and Tomporowski, 2010). However, under conditions of extreme stress, such as when police officers encounter life threatening situations, performance may be impacted in various ways, some of which can be detrimental to performance (e.g., Westmoreland and Haddock, 1989; Artwohl and Christensen, 1997; Morrison and Vila, 1998; Klinger, 2006).

When considering performance generally, maladaptive stress arousal can result in increased task errors and degradation of task accuracy (Driskell and Salas, 1996). These adverse effects primarily involve cognitive functions, such as attention, perception, and decision-making (Driskell and Salas, 1996). Attention is a limited capacity resource, in that only a certain amount of information-processing capacity exists, making it difficult to focus attention on two things at the same time (Vickers, 2007). When attending to a threat, less attention is available for cognitive processing and cognitive overload is more likely to occur, which can result in inattentional blindness (Eysenck et al., 2007; Chabris et al., 2011; Nieuwenhuys and Oudejans, 2011a). Similarly, higher levels of arousal are associated with perceptual narrowing (e.g., tunnel vision, auditory exclusion) because the perceptual field tends to shrink under stress (Vickers, 2007; Honig and Lewinski, 2008). These attentional and perceptual deficits mean that individuals can miss relevant cues (e.g., a subject dropping their weapon; Easterbrook, 1959; Vickers, 2007) and be unable to recall aspects of a situation (Yuille et al., 1994; Hope et al., 2016). Maladaptive stress arousal is also associated with hypervigilant decision-making, which is often impulsive, disorganized, and inefficient (Johnston et al., 1997). Accordingly, Keinan et al. (1987) found that under threat of shock in a laboratory setting, participants completing a computer task tended to offer solutions prior to assessing all alternatives, abandoning their systematic approach of scanning relevant decision options. Research has also demonstrated that police decisions and behaviors, including aggression, during training were found to be associated with maladaptive heart rate (HR) arousal rather than situational factors presented in the scenario (Haller et al., 2014).

Perceptual-motor performance is also degraded by stress, although not to the same extent as cognitive performance (Staal, 2004; Nieuwenhuys and Oudejans, 2011a). For example, a study examining the execution of arrest and self-defense skills demonstrated that under stress, officers were less able to inhibit threat-related processing (e.g., perceptual narrowing) and achieve task-relevant processing (e.g., attentional control), thus leading to poorer task performance (Renden et al., 2014). In line with the default survival response, fine motor skills, such as manipulating a firearm, also tend to be at greater risk for impairment under stress than gross motor skills, such as running (Staal, 2004).

Several studies have examined officer-involved shootings (OIS) to determine how stress may have impacted performance in naturalistic settings. The findings are consistent with the broader stress and performance research. For example, hit rates in annual firearms requalification on the range are near 90% (Anderson and Plecas, 2000), but deteriorate rapidly in the real-world (i.e., hit rates ranging from 14 to 38%; Morrison and Vila, 1998; Morrison and Garner, 2011; Donner and Popovich, 2018). Moreover, under such conditions, officers can experience perceptual distortions, reduced motor dexterity, and impaired cognitive function (e.g., Honig and Sultan, 2004; Artwohl, 2008; Klinger and Brunson, 2009). Artwohl (2008), for example, had 157 police officers complete a survey within a few weeks of being involved in an OIS to examine perceptual and memory distortions that they may have experienced during the high stress incident. The results indicated that the majority of officers experienced perceptual narrowing (i.e., 84% experienced diminished sound and 79% experienced tunnel vision). Most participants (74%) also reported that they responded with little or no conscious thought (i.e., automatic pilot) and many (52%) reported memory distortions or loss. Approximately 7% of the sample reported temporary paralysis, though the author indicated that this may be related to the fleeting freeze response when startled (see LeDoux, 2003), which seems prolonged in high-stress shooting conditions (i.e., 62% reported slow motion time). Similar reactions have been reported in other studies as well (e.g., Honig and Sultan, 2004; Klinger and Brunson, 2009). These effects can be particularly detrimental during a critical incident, when officers are expected to demonstrate sound judgment, proficient performance, and provide accurate recall of their actions.

Manipulating stressful real-world encounters for research purposes would be unethical (Giessing et al., 2019); thus, much of the knowledge that exists today about the physiological impact of stress on performance among police officers come from scenario-based experiments. For example, several studies have found that high stress and anxiety scenarios resulted in impairments to shooting performance (Nieuwenhuys and Oudejans, 2010; Taverniers and De Boeck, 2014; Landman et al., 2016a), quality of skill execution (Renden et al., 2014, 2017; Nieuwenhuys et al., 2016), proportionality of force applied (Nieuwenhuys et al., 2012; Renden et al., 2017), memory (Hope et al., 2016), and communication (Renden et al., 2017; Arble et al., 2019). However, recent studies on police officers demonstrate that the impact of acute stress on performance is complex. For example, stress appears to have differential effects on cognition and physical movement in that rehearsed and automated skills are influenced to a lesser degree (Vickers and Lewinski, 2012; Renden et al., 2017; Arble et al., 2019). Experimental research with simulations is extremely important to not only draw conclusions about what ‘might’ happen to performance in real-world stressful encounters, but also to inform police training to improve public and police safety (Giessing et al., 2019).

While there is no single “best tool” for measuring stress, real-world demands outline the choice of appropriate measures given situational and environmental constraints. Common measures of reactivity to stress capture SNS and HPA axis activation and PNS suppression. Heart rate variability (HRV) is thought to capture changes in the balance between SNS and PNS activity (Thayer et al., 2012), and salivary cortisol is used to capture HPA anticipation and reactivity to stress (Hellhammer et al., 2009). However, during real-world police encounters these measures are highly sensitive to movement (i.e., HRV) or cumbersome to collect without confounds, such as time of day (i.e., salivary cortisol), rendering these methods inappropriate for continuous monitoring throughout police active duty shifts (Dickerson and Kemeny, 2004; Smyth et al., 2013). Current research specifically discourages the collection of HRV while participants are moving because data is highly inconsistent, erroneous, and may lead to false conclusions (Heathers and Goodwin, 2017). Alternatively, HR averaged across time, while controlling for movement, is a robust, ecologically valid, objective, and easily obtainable proxy measure for stress among highly active participants (Vrijkotte et al., 2000).

Previous research supports the feasibility of measuring the stress reactions of officers using HR as they complete their operational duties. Anderson et al. (2002) fitted 76 officers with HR monitors, which were worn prior to and during shifts, and had research assistants record their actions on a minute-by-minute basis during ride-alongs. The results provided HR profiles for various activities. For example, HR became elevated on average to 99–124 beats per minute (bpm; i.e., 40–65 bpm above resting rate) when involved in a use-of-force (UoF) encounter (e.g., physical control, fight, hand on pistol) with a suspect, with maximum HRs reaching 112bpm above resting rate. Similarly, Andersen et al. (2016b) monitored tactical officers during 11 active duty shifts. Researchers matched activities from the officers’ shift notes with their physiological profiles. Study observations revealed that active duty tactical officers operated, on average, at 146 bpm, and ranged from 160 to 180 bpm during UoF incidents, such as pointing a firearm at suspect and warrant executions (Andersen et al., 2016b). Taking a novel approach, Hickman et al. (2011) conducted a pilot study where one officer wore a Garmin global positioning system (GPS)-enabled wrist-watch equipped with a HR monitor. Using GPS data and information from the calls the officer responded to, HR could be visually mapped and associated to specific aspects of CFS. For example, the officer’s heart rate spiked to 165 bpm (69 bpm higher than the officer’s average HR throughout the shift) when conducting a high risk vehicle takedown (i.e., firearm drawn) of an impaired hit-and-run driver who failed to stop for police.

In the current study, continuous ambulatory cardiovascular reactivity was measured on multiple active duty shifts. This was done to develop a “profile” of physiological responses associated with various aspects of police encounters that may influence call outcome. Specifically, this novel approach mapped autonomic stress responses to both the phase of a call (e.g., dispatch, enroute) and incident factors (e.g., call priority, UoF). Advancing previous research, this study utilized GPS and detailed operational police records (e.g., police notes, dispatch records) as objective evidence of specific activities throughout a CFS to be cross-referenced with cardiovascular reactivity data. Furthermore, physical movement (i.e., location and inertia) was tracked and assisted in differentiating whether cardiovascular reactivity was due to physical or psychological stress. It has been argued that, as moderators, experience and training can serve to ‘intervene’ immediately following the presence of a stressor (i.e., blunting the stress response due to previous exposure) or after the stress response occurs (i.e., through the threat appraisal process; Driskell and Salas, 1996; Kavanagh, 2005; Wollert et al., 2011). Results from a UoF simulation study provided some evidence for this moderating effect, with officers on a specialized arrest unit displaying lower HR during a high-pressure scenario, compared to general duty officers (Landman et al., 2016b). Accordingly, individual variables, including an officer’s years of service and training profiles, were examined to conduct a preliminary exploration of whether experience and relevant operational skills training impacted cardiovascular reactivity. Together, these data will provide foundational evidence of what CFS factors are associated with physiological stress responses and to what degree and frequency. This is an important investigation because maladaptive stress responses may be associated with short-term performance impairments (Driskell and Salas, 1996; Nieuwenhuys and Oudejans, 2011a) and long-term health outcomes (Chopko and Schwartz, 2012; Violanti, 2014).

With the use of HR as an indicator of physiological arousal, we tested whether officers’ cardiovascular reactivity uniquely varied as a function of call priority, the phases of a call, incident factors, demographics, experience, and training. We hypothesized the following:

Hypothesis 1: Officers’ cardiovascular reactivity would increase throughout the phases of a call (e.g., from dispatch to encounter).

Hypothesis 2: CFS dispatched with a higher priority level (i.e., very urgent), that involved an arrest/apprehension, UoF, and/or a weapon being reported or accessible, would result in officers experiencing elevated physiological arousal.

Hypothesis 3: Officers with more experience (i.e., years of service) would experience lower cardiovascular reactivity during CFS.

Hypothesis 4: Officers with more relevant operational skills training would experience lower cardiovascular reactivity during CFS.

## Materials and Methods

### Participants

Over a period of nine days, 69 active duty frontline police officers from a large Canadian police agency volunteered to participate in our study. The inclusion criteria for participants were that they were considered ‘fit for duty’ by their police agency and currently on active duty. Screening for diseases was based on self-report. As this is not a diagnostic clinical study, we did not perform medical examinations, however, we did examine self-reported diseases in relation to the data. One participant reported cardiovascular disease and another reported being on medication that affects HR, but their cardiovascular measures (i.e., HR_rest_, HR_average_, and HR_max_) did not significantly differ from other participants and they were thus retained in the study.

A total of 125 shifts were recorded. Data from nine shifts were unusable because the HR data was corrupted (*n* = 3, 2.4%), the HR monitor became dislodged (*n* = 3, 2.4%), or the officer did not respond to any CFS (e.g., scene security; *n* = 3, 2.4%). This resulted in a final sample size of 64 officers over 116 shifts. Over a third of the officers (*n* = 25, 39.1%) participated during one shift, while a large number participated in two (*n* = 29, 45.3%) or three shifts (*n* = 8, 12.5%). One officer participated during four shifts and another during five. In total, approximately 1,200 h of recording time captured HR data for 754 participant responses to 593 CFS. Accordingly, almost a quarter of the CFS (*n* = 142, 23.9%) involved a response from multiple participants.

Table 1 shows the basic sociodemographic characteristics of the sample (*n* = 64). The majority of participants were male (79.7%) and had an average age of 31 years (*SD* = 6.4). Most (87.5%) had obtained post-secondary education. All of the participants were general duty constables with between 1 month and 12 years of service (*M* = 2.06 [years], *SD* = 2.08). Over a quarter (27%) of the participants had previous experience with another law enforcement agency or the military. Training records indicated many participants had received agency training on the conducted energy weapon (CEW; 60.9%), carbine (73.4%), and responding to active threats (81.3%). There were five participants (7.9%) who reported having been involved in a lethal force encounter, as either the officer discharging their firearm, or a witness officer on scene.

**TABLE 1:** Participant demographics.

**Demographic factors**	***n***	**%**	***M***	***SD***
Sex				
Male	51	79.7%		
Female	13	20.3%		
Age	64		31	6.4
Height (in)	64		73	13.8
Weight (lb)	64		182	28.6
Highest level of formal education				
High school diploma or equivalent	8	12.5%		
Registered Apprenticeship or other trades certificate or diploma	2	3.1%		
College or other non-university certificate or diploma	18	28.1%		
University certificate or diploma below bachelors level	12	18.8%		
Bachelors degree	20	31.3%		
Post graduate degree above bachelors level	4	6.3%		
Current rank				
Constable	64	100.0%		
Current duty type				
General duty	64	100.0%		
Years of service with the agency	64		2.06	2.08
Prior service with another police agency or the military	17	27.0%		
Training experience				
Instructor experience in the area of use-of-force	3	4.8%		
Specialized training in the area of use-of-force (outside of the agency)	15	23.8%		
Martial arts	23	36.5%		
Active shooter	52	81.3%		
Conducted energy weapon	39	60.9%		
Carbine	47	73.4%		
Involved in a lethal force encounter	5	7.9%		

### Materials

#### Demographics and Shift Questionnaires

A short demographics questionnaire was used to collect age, gender, years of service, law enforcement experience, and training. A pre-shift questionnaire was used to collect basic information on general health factors (e.g., exercise, sleep), while the UoF and level of fatigue during the shift were captured with a post-shift questionnaire.

#### Operational Police Records

Operational police records were obtained and reviewed to categorize officers’ activities throughout their shift. Operational records included: (1) police notes, which are typically prepared during or shortly after a police occurrence and are used by officers as an aide memoire for court purposes; (2) occurrence files, which are created for the officer(s) to add reports (e.g., general, supplemental) and outline details concerning the circumstances of the call, individuals involved, actions taken, and whether charges were laid; (3) UoF reports, which an officer completes to articulate the use of an intervention and describe the officer’s risk assessment; and (4) computer-aided dispatch (CAD) records, which provide time-stamped radio communications (e.g., contact with subject, arrest), officer status (e.g., dispatched, enroute, on scene), and messages to mobile workstations.

#### Monitoring Devices

HR, GPS, and physical movement were captured with a Polar V800 watch, H7 chest strap HR sensor, and Stride sensor, which is a foot mounted inertia sensor (Polar Electro Oy, Kempele, Finland). The H7 is paired through Bluetooth with the Polar V800 to record cardiovascular reactivity at one second intervals. Polar HR monitors are regularly used to measure HR in police research (Barton et al., 2000; Anderson et al., 2002; Meyerhoff et al., 2004; Hulse and Memon, 2006; Hope et al., 2012; Kayihan et al., 2013; Renden et al., 2015; Hope et al., 2016; Landman et al., 2016a) and the technology has been validated against electrocardiograms (ECG; Gamelin et al., 2006; Nunan et al., 2008, 2009; Weippert et al., 2010; Quintana et al., 2012; Wallén et al., 2012; Giles et al., 2016; Barbosa et al., 2016). The Polar V800 is equipped with an integrated GPS that tracks speed (kilometers per hours; km/h), pace (min/km), cadence (steps/min), distance (m), location (latitude and longitude), and route. The Stride sensor automatically calibrates with the V800’s GPS to capture more accurate and detailed physical movement. The battery duration of the V800 is up to 13 h with continuous GPS recording, which covers the typical police shift.

### Measures

#### Heart Rate

Consistent with previous research, the HR monitors attached to officers were used to collect several measures of cardiovascular reactivity: resting HR during the shift (HR_rest_), maximum HR (HR_peak_) reached during each phase of the call (see below for more details), and average heart rate throughout the shift (HR_average_) (Anderson et al., 2002; Andersen and Gustafsberg, 2016; Andersen et al., 2016b). HR_rest_ is best determined immediately upon waking in the morning, as HR measures taken before or during a shift may include anticipatory stress regarding the upcoming shift or potential events that might be encountered during the current shift and therefore be slightly higher than actual resting HR (Plowman and Smith, 2013). Resting HR can also be affected by body position and is reported to be higher when sitting, as opposed to when lying supine (Miles-Chan et al., 2013). However, for logistical reasons, HR_rest_ in this study was based on the lowest 1-min HR while an officer was on shift. Similar methods for determining HR_rest_ have been used in previous research (Anderson et al., 2002; Andersen and Gustafsberg, 2016). HRrest during sleep was collected for a small subsample (*n* = 10) for comparative purposes. HR_max_ and HR_min_ represented the highest and lowest HR during the officer’s shift. To provide a standardized measure for between-subject analysis, the difference (HR_peak above resting_) between the officers’ HR_peak_ during phases of the call and their HR_rest_ was calculated (Anderson et al., 2002; Andersen et al., 2016b).

#### Movement

Speed (km/h), which was captured by the GPS and the inertia sensor, was collected to control for physical movement throughout an officer’s shift. This assisted us in determining whether cardiovascular reactivity resulted from physical or psychological stress. For example, a large increase in HR absent of physical movement would suggest a psychological stress response. For reference purposes, average walking speed is approximately 5 km/h (Bohannon, 1997). A slow or average jogging pace is 8 km/h and a fast jog is 11 km/h (Schnohr et al., 2015). Research on law enforcement cadets has also found that average sprint speeds are approximately 23 1/2 km/h (Lewinski et al., 2015; Crawley et al., 2016).

#### Phase of the Call

**FIGURE 1:**
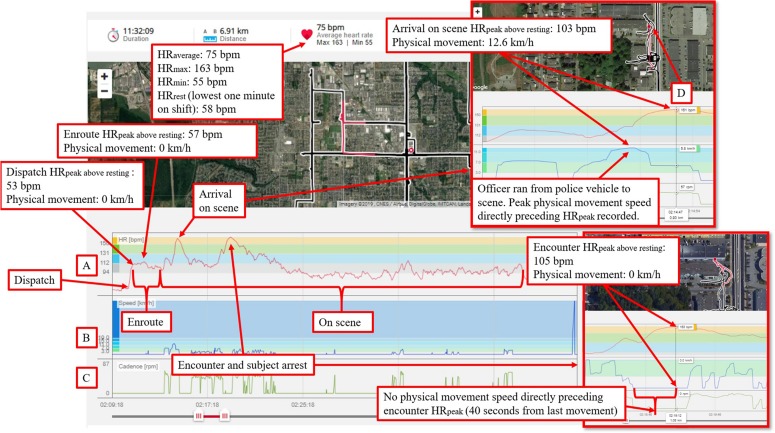
A disturbance CFS provides a graphical representation of how stress-activity mapping was conducted using HR, GPS, and inertia data in Polar Flow. The left vertical axis **(A)** presents HR (bpm), in red on the horizontal axis. The left vertical axis **(B)** presents speed (km/h) from the GPS, in blue on the horizontal axis. The left vertical axis **(C)** presents cadence [rpm] from the Stride sensor, in green on the horizontal axis. A tracking meter **(D)** identifies HR and movement measures at a specific point in time, which is linked to the corresponding GPS position on the map. Physiological data can be highlighted and zoomed in for detailed examination. Imagery 2019, CNES/Airbus, DigitalGlobe, IMTCAN, Landsat/Copernicus, McElhanney.

Using GPS data and operational police records, HR_peak_ and movement were broken down temporally into four phases of the call: (1) dispatch, (2) enroute, (3) arrival on scene, and (4) encounter, UoF and/or arrest (see Figure 1). The first three phases were determined using GPS data and officer status timestamps from the CAD (e.g., dispatched, enroute, on scene). The fourth phase was established by cross-referencing GPS data, inertia sensor data, time-stamped radio communications (e.g., contact with subject, arrest), officer notes, occurrence files, and UoF reports.

#### Incident Factors

CFS were classified based on dispatch priority levels (1 through 3). To ensure CFS are dispatched in a consistent manner, dispatchers adhere to standard operating procedures to assign priority levels. Priority levels are defined as:

Priority 1 – Very Urgent – Immediate Dispatch. A major incident or incident in progress that requires immediate police presence, assistance or service. Involves the report of a loss of life or a need for police to prevent a loss of life.

Priority 2 – Urgent – Dispatch as soon as possible. There is an urgent need for police presence, assistance or service. While there is no loss of life involved, the potential for escalation of violence exists.

Priority 3 – Routine – Dispatch as soon as reasonably possible. Reports that do not require immediate police presence, assistance or service.

From the post-shift questionnaires and operational records, CFS were also coded for whether weapons were reported or accessible (0 = no, 1 = yes) during any phase of a call, if there was an arrest or apprehension (0 = no, 1 = present while other officer conducted arrest, 2 = *Mental Health Act* apprehension, 3 = arrest), and whether the encounter involved UoF (0 = no, 1 = non-firearm, and 2 = firearm). UoF included the use of physical control techniques, both soft (e.g., joint locks, soft takedowns) and hard (e.g., stuns and strikes, hard takedowns), less lethal options (e.g., CEW), and firearms, with or without a subject present. For example, clearing an empty building with a firearm drawn was categorized as UoF.

#### Training

Officers’ training records and the training information captured in the demographics form were used to identify the following six experience criteria: (1) instructor experience in the area of UoF, (2) specialized training in the area of UoF (outside of the agency), (3) martial arts, (4) active shooter, (5) CEW, and (6) carbine. All officers had taken the agency’s mandatory crisis intervention and de-escalation training. To create a composite training variable, the sum of the training experience criteria for each officer was calculated. A score was assigned to each participant to indicate the number of experience criteria the officer had (0 = least and 6 = most; see Table 2). While the categorization does not take into account the recency and frequency of training experience, nor weight types of training differently, it provides a basic measure that enabled us to examine the effect of training on cardiovascular reactivity during CFS.

**TABLE 2:** Composite training score, indicating the number of experience criteria the officer possessed (0 = least and 6 = most).

**Level of training**	***n***	**%**
1	9	14.1%
2	13	20.3%
3	29	45.3%
4	9	14.1%
5	3	4.7%
6	1	1.6%
Total	64	100.0%

#### Procedure

To improve the likelihood of capturing physiological responses to high-stress encounters, the selection of the study location and collection period were informed by an examination of UoF trends and violent crime severity indexes in Canadian cities. The urban city that was selected had approximately 700 operational officers and five policing districts. The two districts that were targeted have a population of approximately 220,000 and an area of 86 km^2^. Work shifts were 12 h in length with staggered start times. Early morning shifts started at 0600 h and late morning shifts started at 0930 h. Early night shifts started at 1700 h and late-night shifts start at 1900 h. Participants were recruited by having the District Watch Commanders send a callout message via internal e-mail. Researchers also recruited at the pre-shift briefings.

Those interested in participating in the study completed a written informed consent form and were asked to take standard notes throughout their shift, indicating the time and call for service/activity that they were involved in. Participants were then equipped with a Polar V800 watch, H7 chest strap HR sensor, and Stride sensor. Following this, officers completed a demographics and pre-shift questionnaire. Monitoring devices were worn for the entirety of their shift. At the end of their shift, recordings were stopped, equipment removed, and the officers then completed a post-shift questionnaire. A copy of each officer’s notebook notes for the shift were obtained. Each participant received a debriefing form and $50 financial compensation. A small subsample (*n* = 10) volunteered to wear a HR monitor during their normal sleep cycle so that we could obtain their resting HR while sleeping. These participants received an additional $50 in financial compensation.

After the field work was completed, the researchers accessed operational files, UoF reports, dispatch logs for the CFS, as well as training profiles for all the participants. Anonymized HR, GPS, and Stride sensor data were uploaded to the Polar Flow web application (Polar Electro Oy, 2016) where they were integrated with maps and charts for visual analysis and coding (see Figure 1). All procedures were approved by Carleton University’s Research Ethics Board (REB #17-106853) and the agency’s Research Review Board (RRB).

### Data Analyses

Data from the stress-activity mapping (i.e., officer HR and movement data for corresponding phases of the call), along with incident factors and demographic data, were entered into SPSS v.22 (IBM Corp, 2013, Released) for quantitative analysis. All data were checked for expected ranges, presence of outliers and abnormal values. The Shapiro–Wilk test was used to assess normality (no assumptions were violated). The descriptive data are presented as frequencies, rates (%), means, and standard deviations. Paired-samples *t*-tests are used to test the mean difference between paired observations. The reported statistical tests are one-tailed, and the significance value is set to *p* < 0.05. Descriptive statistics for HR_peak above resting_ across CFS are reported for each phase of the call as a function of incident factors (e.g., call priority).

To examine how the standardized measure of cardiovascular reactivity (HR_peak above resting_) varied as a function of the phases of the call, demographics, incident factors, and training, linear mixed models (LMM) for repeated measures are used. LMM is a flexible approach for the analysis of repeated measures data and has several advantages over traditional methods (e.g., ANOVA). LMM can appropriately handle missing data and therefore does not exclude cases with a missing time point (Gueorguieva and Krystal, 2004). Moreover, the LMM can account for uneven spacing and correlation between repeated measurements on the same subjects and does not assume homogeneity of variance across groups and time points (Gueorguieva and Krystal, 2004; Blackwell et al., 2006). Time-varying covariates may also be included in the LMM (Blackwell et al., 2006); allowing for speed (km/h) at each phase of the call to be used as a covariate to control for movement. The LMM model will use a two-level hierarchical data structure: CFS as level-1 and participants as level-2. The model will include a random intercept to accommodate correlations in the outcome variables across CFS for each participant. All other predictors and covariates, including phase of the call, were specified as fixed effects. To compare fixed effects across models, maximum likelihood (ML) estimation was used (Zuur et al., 2009). The Bonferroni correction was used as a *post hoc* test to control for type I errors.

## Results

### Shift HR

Table 3 presents HR data for officers across their shifts. A subsample (*n* = 10) wore a HR monitor to sleep to obtain an off-shift resting heart rate for comparative purposes. A paired-samples *t*-test was conducted to compare HR_rest_ at the lowest 1 min while on shift and while the officer was sleeping. There was a significant difference in HR_rest_ at the lowest 1 min while on shift (*M* = 64.60, *SD* = 6.74) and while the officer was sleeping (*M* = 55.40, *SD* = 6.60), *t*(9) = −4.261, *p* = 0.001, *d* = 1.35. Therefore, as expected, the resting rate in this study was slightly higher than actual resting HR during sleep. This may be attributed to factors such as anticipatory stress while on-shift or the officer’s body positioning during the recording (e.g., sitting in police vehicle). As such, HR_rest_ in this study reflects the realities of an officer being at rest while on-shift and provides a context relevant baseline measure to standardize increases in HR (HR_peak above resting_) between the officers.

**TABLE 3:** Descriptive statistics for officer HR across shifts.

	***N***	***M***	***SD***	***Min***	***Max***
HR_rest_	64	63.1	9.7	40.0	85.0
HR_average_	64	82.7	10.7	54.0	102.4
HR_min_	64	59.3	8.8	38.0	81.0
HR_max_	64	147.6	19.6	109.0	203.0

*HR_*rest*_ is the lowest one minute on shift.*

### Call for Service

The types of calls responded to by participants, along with their associated dispatch priority level, are presented in Table 4. Disturbances (9.3%) and abandoned 911 calls (8.9%) were the most common calls that participants responded to. CFSs were most frequently dispatched as urgent (*n* = 524/754, 69.5%), followed by routine (*n* = 171/754, 22.7%), and very urgent (*n* = 59/754, 7.8%). Calls for weapons, shots fired, and assaults in progress were most commonly dispatched as very urgent.

**TABLE 4:** Frequency of call type by priority level.

**Call type**	**Priority level**				
	**1 – Very**	**2 –**	**3 –**		
	**Urgent**	**Urgent**	**Routine**	**Total**	
		
	***n***	***n***	***n***	***n***	**%**
Disturbance	3	65	2	70	9.3%
Abandoned 911	4	58	5	67	8.9%
Check wellbeing	0	53	1	54	7.2%
Bylaw	0	4	35	39	5.2%
Domestic in progress	7	23	0	30	4.0%
Assault report	0	26	3	29	3.8%
Assist police/fire/ambulance	0	26	2	28	3.7%
Alarm	0	27	0	27	3.6%
Unwanted person	0	14	11	25	3.3%
Suicidal person	5	19	0	24	3.2%
Fight	2	20	0	22	2.9%
Weapon	12	9	0	21	2.8%
Assist general public	0	9	12	21	2.8%
Suspicious person	0	14	4	18	2.4%
Motor vehicle incident	0	16	2	18	2.4%
Drugs	0	12	5	17	2.3%
Suspicious circumstances	1	12	3	16	2.1%
Suspicious vehicle	0	7	7	14	1.9%
Mischief	0	4	10	14	1.9%
Threats	0	5	7	12	1.6%
Shots fired	9	0	0	9	1.2%
Break end enter in progress	0	9	0	9	1.2%
Theft of vehicle	0	3	5	8	1.1%
Theft in progress	0	8	0	8	1.1%
Assault in progress	7	1	0	8	1.1%
Animal	0	6	2	8	1.1%
Other call types (<1%)	9	74	55	138	18.3%
Total	59	524	171	754	100.0%

### Stress Reactivity

To examine the participant’s cardiovascular reactivity during CFS, descriptive statistics for HR_peak above resting_ as a function of incident factors (e.g., call priority) and phases of the call (e.g., dispatch) are displayed (see Table 5). Average HR_peak above resting_ was lowest during the dispatch phase (*M* = 25.94, *SD* = 13.62), and increased while enroute (*M* = 32.50, *SD* = 13.42), and when arriving on scene (*M* = 46.37, *SD* = 16.32). Average HR_peak above resting_ was highest during the encounter/UoF/arrest phase of the call (*M* = 55.30, *SD* = 20.25). Throughout all phases of the call, average HR_peak above resting_ increased with the urgency of the priority level and the report or accessibility of a weapon(s) (*n* = 43/754, 5.7%). As expected, arrest (*n* = 68/754, 9%) and apprehension (*n* = 26/754, 3.4%) of a subject resulted in more pronounced increases in average HR_peak above resting_ during the latter phases of the call, compared to the earlier phases. As the level of force increased from none, to non-firearm (*n* = 71/754, 9.4%), to firearm (*n* = 27/754, 3.6%), average HR_peak above resting_ also increased. Interestingly, elevated average HR_peak above resting_ can be observed during all phases of the call when force was used. For example, incidents where officers drew their firearm were those with the highest average HR_peak above resting_ during dispatch (*M* = 38.5, *SD* = 20.4), while enroute (*M* = 44.2, *SD* = 21.1), when arriving on scene (*M* = 57.9, *SD* = 18.7), and during the encounter (*M* = 67.5, *SD* = 14.5).

**TABLE 5:** HR_peak above resting_ as a function of incident factors and phases of the call.

													**Encounter/use-of-**			
**Incident factors**	**Dispatch**				**Enroute**				**Arrival on scene**				**force/arrest**			
	**HR_peak above resting_^a^**				**HR_peak above resting_**				**HR_peak above resting_**				**HR_peak above resting_**			
	**(*n* = 741)**				**(*n* = 697)**				**(*n* = 681)**				**(*n* = 272)**			
	***M***	***SD***	***n***	**%**	***M***	***SD***	***n***	**%**	***M***	***SD***	***n***	**%**	***M***	***SD***	***n***	**%**
**Call priority**																
(1) Very urgent	32.7	15.2	59	8.0%	39.5	18.5	54	7.7%	53.1	20.5	52	7.6%	56.9	25.3	22	8.1%
(2) Urgent	26.0	14.0	518	69.9%	33.0	13.3	490	70.3%	46.4	15.9	475	69.8%	55.5	19.7	207	76.1%
(3) Routine	23.2	10.8	164	22.1%	28.5	9.9	153	22.0%	44.1	15.4	154	22.6%	53.6	20.6	43	15.8%
**Weapon(s) reported/accessible**																
Yes	36.3	16.4	42	5.7%	39.9	20.4	43	6.2%	54.2	19.7	40	5.9%	55.5	24.7	26	9.6%
No	25.3	13.2	699	94.3%	32.0	12.7	654	93.8%	45.9	16.0	641	94.1%	55.3	19.8	246	90.4%
**Arrest/apprehension**																
Arrest	29.7	14.2	63	8.5%	38.8	16.8	66	9.5%	58.3	17.0	66	9.7%	62.9	24.5	63	23.2%
MHA apprehension	30.2	16.8	26	3.5%	32.9	14.6	25	3.6%	50.5	16.2	25	3.7%	55.0	26.3	23	8.5%
Present while other officer conducted arrest	29.0	13.1	21	2.8%	38.3	17.1	21	3.0%	51.7	12.1	21	3.1%	46.4	13.1	19	7.0%
No	25.3	13.4	631	85.2%	31.6	12.6	585	83.9%	44.6	15.8	569	83.6%	53.5	17.3	167	61.4%
**Use of force**																
Firearm	38.5	20.4	27	3.6%	44.2	21.1	27	3.9%	57.9	18.7	27	4.0%	67.5	14.5	25	9.2%
Non-firearm	29.0	15.4	68	9.2%	37.9	17.0	69	9.9%	55.6	16.4	70	10.3%	63.2	26.4	63	23.2%
No	25.1	12.8	646	87.2%	31.3	12.1	601	86.2%	44.7	15.6	584	85.8%	50.9	16.8	184	67.6%

*Sample size decreases across phases of the call, since for various reasons, not all dispatched officers travel to the scene, attend the scene, and/or come into contact with the subject of the call (e.g., officer called off, subject gone upon officer’s arrival). ^*a*^Resting heart rate was 63 beats per minute.*

To examine how cardiovascular reactivity (HR_peak above resting_) varied as a function of the phases of the call, demographics, incident factors, and training, the results of the LMM for repeated measures are presented (see Table 6). Two models are displayed: one with speed (km/h) at each phase of the call as a covariate to control for movement and one without movement. Results for the model without movement will be presented and contrasted when differences appear in the model including movement. Based on this sample of officers and CFS, estimates (*B*) from the models (see Table 6) can be used to approximate average stress reactivity experienced by officers during CFS. For example, the following formula can be developed for a male officer responding to a priority 1 call with a weapon reported, where the officer clears a residence with his firearm drawn (at an average walking speed – 5 km/h), resulting in the location and arrest of a subject:

**TABLE 6:** Linear mixed-effects model for repeated measures with HR_peak above resting_ as a function phases of the calls, officer characteristics, incident factors, and training with and without movement as a covariate.

	**Without movement**				**With movement**			
	***B***	***SE***	***t***	***p***	***B***	***SE***	***t***	***p***
Fixed effects								
(intercept)	28.61	5.40	5.30	< 0.001	28.66	5.23	5.48	< 0.001
Phase of call^a^								
Encounter/use-of-force/arrest	24.39	1.04	23.52	< 0.001	18.15	1.02	17.72	< 0.001
Arrival on scene	20.28	0.65	31.36	< 0.001	11.58	0.67	17.32	< 0.001
Enroute	6.41	0.45	14.11	< 0.001	7.33	0.40	18.20	< 0.001
Dispatch	–				–			
Sex								
Female	3.39	2.21	1.54	0.130	2.71	2.14	1.27	0.209
Male	–				–			
Age	–0.18	0.16	–1.12	0.266	–0.21	0.15	–1.35	0.181
Years of service	–0.08	0.47	–0.17	0.867	0.03	0.46	0.07	0.942
Call priority								
(1) Very urgent	7.00	1.55	4.52	< 0.001	5.61	1.39	4.04	< 0.001
(2) Urgent	1.82	0.87	2.09	0.037	1.32	0.78	1.68	0.093
(3) Routine	–				–			
Weapon(s) reported/accessible								
Yes	3.80	1.60	2.37	0.018	4.11	1.44	2.85	0.005
No	–				–			
Arrest/apprehension								
Arrest	6.48	1.74	3.72	< 0.001	6.49	1.57	4.14	< 0.001
MHA apprehension	3.65	2.02	1.80	0.072	2.80	1.81	1.54	0.123
Present while other officer conducted arrest	3.17	2.19	1.45	0.148	1.47	1.97	0.75	0.454
No	–				–			
Use-of-force								
Firearm	8.30	1.90	4.36	< 0.001	7.64	1.71	4.46	< 0.001
Non-firearm	0.58	1.77	0.33	0.743	–0.17	1.59	–0.11	0.913
No	–				–			
Level of training	–0.22	0.81	–0.28	0.784	–0.19	0.78	–0.24	0.807
Movement	–	–	–	–	2.53	0.11	24.04	< 0.001
**Random effects**	**Var**	***SD***			**Var**	***SD***		
Officer (intercept)	36.47	6.0			35.69	6.0		

*Unstandardized regression coefficient (*B*), standard error (*SE*), and *t-*value (*t*). Mean resting heart rate was 63 beats per minute. ^*a*^Repeated measures.*

146 bpm (estimated HR) = HR_rest_ (63.1) + intercept (28.66) + priority 1 (5.61) + weapon reported/accessible (4.11) + average walking speed (5 km/h × 2.53) + officer use of firearm (7.64) + encounter phase (18.15) + arrest (6.49).

#### Phase of the Call

**FIGURE 2:**
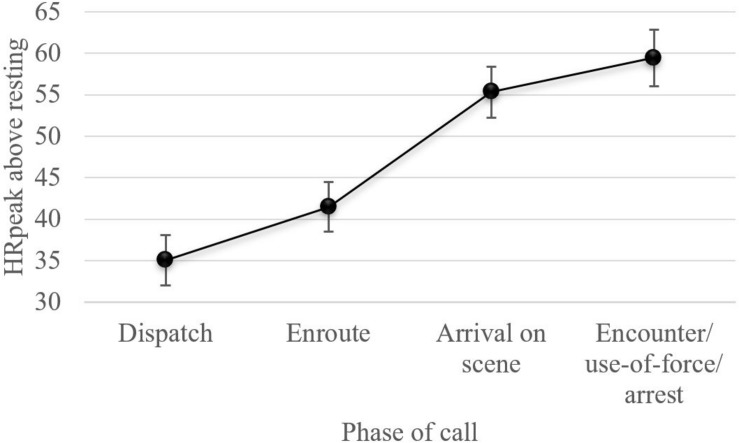
Line chart displaying estimated marginal means for phase of call, without movement as a covariate, from the linear mixed-effects model for repeated measures (of note, results did not significantly change when movement was included as a covariate – see Table 6). HR_rest_ was 63 bpm.

Our first hypothesis, that officers’ cardiovascular reactivity would increase throughout the phases of the call (e.g., from dispatch to encounter), was tested using a repeated measures analysis. The repeated measures analysis without speed as a covariate determined that HR_peak above resting_ significantly differed across the phases of the call [*F*(3,567.455) = 384.390, *p* < 0.001]. In support of the hypothesis, the Bonferroni *post hoc* correction revealed that HR_peak above resting_ during the encounter (*M* = 59.46, *SE* = 1.75) was significantly higher than when being dispatched to the call (*M* = 35.07, *SE* = 1.54, *p* < 0.001), while enroute (*M* = 41.48, *SE* = 1.53, *p* < 0.001), and when arriving on scene (*M* = 55.35, *SE* = 1.57, *p* < 0.001). Results remained significant at the *p* < 0.001 level when controlling for movement. See Figure 2 for a line chart of estimated marginal means for phase of call.

#### Incident Factors

Recall that our second hypothesis was that CFS dispatched with a higher priority level (i.e., very urgent), that involved an arrest/apprehension, UoF, and/or a weapon being reported or accessible, would result in officers experiencing elevated physiological arousal. Results for the incident factors show that HR_peak above resting_ significantly differed as a function of call priority [*F*(2,713.764) = 10.221, *p* < 0.001], reported/accessible weapon(s) [*F*(1,690.781) = 5.594, *p* = 0.018], arrest/apprehension [*F*(3,666.173) = 4.884, *p* = 0.002], and UoF [*F*(2,671.957) = 9.5, *p* < 0.001]. Results remained significant when controlling for movement. Specifically, results indicate that very urgent calls were associated with a 7 bpm increase in heart rate compared to routine calls (*p* < 0.001), while the report/accessibility of a weapon(s) increased heart rate by 3.8 bpm (*p* = 0.018). An incident involving an arrest resulted in a 6.5 bpm increase in heart rate, compared to one that did not (*p* < 0.001). Similarly, responses involving a participant’s use of their firearm elevated heart rate by 8.3 bpm compared to a response that involved no UoF (*p* < 0.001). Incidents involving non-firearm UoF did not result in a significant increase in HR (0.58 bpm, *p* = 0.784), however, this can be attributed to the collinearity between arrest/apprehension and UoF (i.e., most arrests involve some level of force, such as soft physical control techniques). In fact, when the arrest/apprehension factor was removed from the model, non-firearm UoF resulted in a 5.6 bpm increase in heart rate (*p* < 0.001). Overall, our hypothesis was supported and, with the exception of call priority, results remained consistent when controlling for movement.

#### Demographics and Experience

Results for the demographic and experience characteristics show that HR_peak above resting_ did not significantly differ as a function of gender [*F*(1,65.255) = 2.216, *p* = 0.141], age [*F*(1,66.842) = 1.259, *p* = 0.266], or years of service [*F*(1,61.406) = 0.028, *p* = 0.867]. Results remained non-significant when controlling for movement. Due to collinearity between age and years of service, models were run that retained one variable, while excluding the other. Neither age (*B* = −0.191, *p* = 0.169) nor years of service (*B* = −0.344, *p* = 0.412) became significant with this approach. The results did not support our third hypothesis, that officers with more experience (i.e., years of service) would experience lower cardiovascular reactivity during CFS.

#### Training

Our fourth hypothesis, that officers with more relevant operational skills training would experience lower cardiovascular reactivity during CFS, was also not supported. In both models, with and without movement, the composite training variable created from the sum of the training experience criteria for each officer had a non-significant effect on HR_peak above resting_ [*F*(1,66.555) = 0.076, *p* = 0.784].

#### Movement

When speed (km/h) at each phase of the call was included as a covariate to control for movement, it had a significant effect on HR_peak above resting_ [*F*(1,1664.088) = 577.717, *p* < 0.001]. Results indicate a 2.5 bpm increase in heart rate for every 1 km/h increase in movement. The inclusion of speed (km/h) in the model did not significantly alter the results of the model, except for estimates of HR during the phase of the calls. Specifically, decreases in estimated HR were observed during the phases of the call where one would expect more movement. Thus, controlling for movement, estimates for arrival on scene and the encounter/UoF/arrest decreased by 8.7 and 6.2 bpm, respectively.

## Discussion

The current study measured continuous ambulatory cardiovascular reactivity to develop a “profile” of physiological responses associated with various aspects of police encounters. This novel approach expanded on the pilot work of Hickman et al. (2011), to establish the feasibility of using GPS and detailed operational police records to map general duty police officers’ autonomic stress responses to the phase of a call and incident factors. Consistent with the findings of Anderson et al. (2002), the current study sample demonstrated that officers had a HRmin of 59 bpm and an HR_average_ of 83 bpm during their shift. The striking similarity between HR measures in our study and those in the only other known study involving on-shift HR tracking of general duty officers, improves the generalizability of our results. The current research also builds on the growing body of evidence (e.g., Anderson et al., 2002; Andersen et al., 2016b) indicating that stress arousal is a real consideration in general duty policing. For example, in our study, significant cardiovascular reactivity was observed during shifts with HR_max_ averaging 148 bpm for participants and ranging up to 203 bpm.

Building on the work of Anderson et al. (2002), our use of advanced statistical methods (i.e., LMM for repeated measures) allowed us to examine how officers’ cardiovascular reactivity uniquely varied as a function of call priority, the phases of the call, incident factors, demographics, and training. Results indicate that very urgent priority 1 calls, which accounted for 8% of CFS in this study, were associated with a 7 bpm increase in HR compared to routine calls (*p* < 0.001). As we hypothesized, independent of incident factors, average HR at dispatch (98 bpm) was significantly higher than HR_rest_ and steadily elevated while enroute (105 bpm), when arriving on scene (118 bpm), and during the encounter/UoF/arrest phase of the call (123 bpm); demonstrating increasing arousal throughout a CFS (see Figure 2). Moreover, in support of our second hypothesis, specific incident factors, such as the report/accessibility of a weapon(s), making arrests, and drawing one’s firearm, increased heart rates (by 3.8, 6.5, and 8.3 bpm, respectively) relative to calls where these factors were not present. Unfortunately, it was not possible to consistently determine the phase of a call that an officer became aware of a weapon (or potential weapon). This limits our ability to tease apart whether the influence of weapons on cardiovascular reactivity presented from a perceived (anticipatory) or real threat.

In the current study, individual variables including an officer’s age, gender, years of service, and training profiles, were examined to conduct a preliminary exploration of whether demographic variables, experience, or relevant operational skills training impacted cardiovascular reactivity. None of these variables showed a significant effect, indicating that physiological arousal may not be a function of officer characteristics, nor the level of experience (i.e., years of service) or the type of training that was examined in this study, as we hypothesized. Instead, as discussed above, stress reactivity was primarily associated with higher risk incident factors. The findings related to experience and training align with studies of tactical officers, who generally respond to high risk encounters (Andersen et al., 2016a, b). Specifically, Andersen et al. (2016a, b) found that tactical officers, despite their many years of service and elite training, typically operate at a higher level of arousal (e.g., 146 bpm), ranging from 160 to 180 bpm during UoF encounters. In both types of research (general duty and tactical officers) we see that typical police training alone does not seem to reduce physiological arousal to high risk calls. Thus, it seems likely that call risk, or perceived call risk, rather than training itself, may be determining an officer’s level of physiological arousal. That being said, it is worth reiterating that although the training results of the current study align with previous research, the basic training measure used in the current study was limited. Specifically, the composite training variable did not consider the recency or frequency of training experience, nor weight types of training differently. Future research should use a more sophisticated measure of training that considers these additional factors.

**FIGURE 3:**
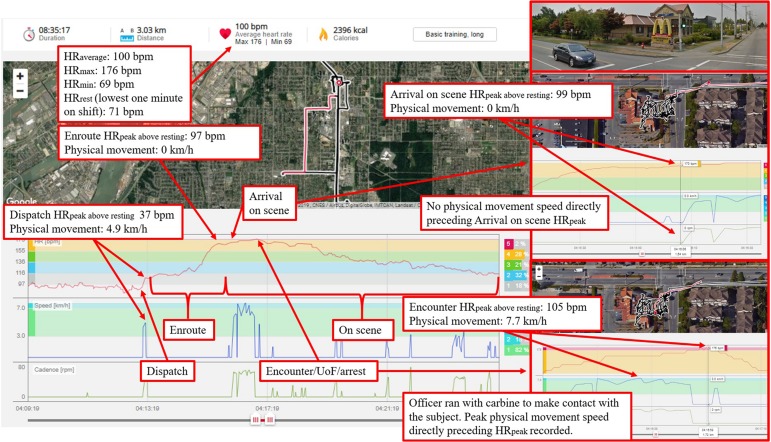
The officer was responding to an assault in progress at a McDonald’s restaurant. While enroute, dispatch advised that the subject was possibly armed with a firearm. When arriving on scene, before leaving the police vehicle, the officer’s HR had reached 170 bpm (absent of movement). The officer, equipped with his carbine, made contact with the subject through the drive-thru window. The subject had a screw driver in one hand and a spatula in the other. The subject complied with commands to drop the weapons and exited from the drive-thru window. The subject was laid face-down on the ground, but then stood up and took an assaultive stance with clenched fists. The officer transitioned from his carbine and deployed his conducted energy weapon in probe mode. The subject was then taken into custody by multiple officers. Imagery 2019, CNES/Airbus, DigitalGlobe, IMTCAN, Landsat/Copernicus, McElhanney.

Notably, this was the first known on-shift policing study to objectively measure physical movement (i.e., location and inertia) to assist in differentiating whether cardiovascular reactivity was due to physical or psychological stress. We know from medical science that movement of the body increases oxygen demands to the muscles and thus could be responsible for the increase in heart rate (via increases in respiration to meet oxygen demands). In this study we were not able to collect respiration rate. Thus, we used movement as a covariate (a proxy of increased oxygen demands) to examine if the increases in heart rate could be explained by purely physical reasons (i.e., oxygen demands to the muscles); if not, then increases in heart rate potentially stem from psychological stress. Interestingly, increases in HR resulting from physical movement appeared to be largely independent of increases in HR related to incident factors (e.g., arrest, use of a firearm). Therefore, significant increases in HR, which were observed when officers were presented with a real or perceived threatening stimulus (i.e., priority 1, reported/accessible weapon[s], arrest/apprehension, and the UoF), appear to be attributable to psychological stress and the initiation of the fight-or-flight response. The inclusion of movement as a covariate in research examining on-shift stress in general duty police encounters is a novel contribution to the field and these results support that psychological stress is a consistent and central component of operational police responses. A real-world example from the study (see Figure 3), demonstrates a case of psychological stress during a high risk CFS.

Overall, the general findings reported above form a foundational step for future research investigating the impact of (psychologically related) physiological arousal. This research is likely to have implications for three important components associated with policing: performance, training, and long-term health.

### Relationship Between Stress, Experience, and Performance

The relationship between arousal, experience, and performance in police encounters is complex and not fully understood. Fortunately, several policing studies have demonstrated that realistic scenarios can be developed that elicit average HR that replicate stressful real-world encounters (i.e., ∼140 bpm or more). These scenarios provide researchers with the opportunity to carefully study the relationship between these various factors. Within these scenarios, stress reactivity can result in perceptual distortions (e.g., tunnel vision, auditory exclusion) as well as increased performance errors and deficits in verbal communication, of the sort that are often witnessed in the field (Meyerhoff et al., 2004; Lewinski, 2008; McCraty and Atkinson, 2012; Brisinda et al., 2015; Andersen and Gustafsberg, 2016; Andersen et al., 2016b; Arble et al., 2019). However, while stress can deteriorate police performance, officer experience and training has been shown to improve performance in UoF scenarios in some studies. Specifically, studies have shown that, compared to novices, experienced and elite officers often demonstrate improved decision-making processes, attentional control, shot accuracy, and cue recognition, as well as fewer decisions errors (Vickers and Lewinski, 2012; Renden et al., 2015; Boulton and Cole, 2016; Landman et al., 2016b).

Given these findings, the interaction between stress, training, and performance requires further examination. For example, it would be important to determine if there is an optimal range of physiological arousal for best performance, whether this optimal range varies as a function of experience and training, and whether this optimal range varies by call type, call priority, and/or call phase. Once these issues have been examined in scenario-based studies, confirming that the results can be replicated in field studies is important. This seems particularly important given the results of the current study, where experience factors (as measured in the current study and discussed above) were not related to stress reactivity. While performance was not examined in our study, we believe that with slight modifications the methods we used could provide the foundation for future research on the relationship between stress, experience, and performance. For example, a ride-along component could be added to assess performance as other researchers have recently done (e.g., Todak and James, 2018).

### Evidence-Based Training

While the body’s default response to successfully cope with a threat is to stimulate fight-or-flight physiology (LeDoux and Pine, 2016), research indicates that this threat response is malleable, with certain types of training being shown to improve performance and increase resilience to stress reactions (Driskell et al., 2001; Arnetz et al., 2009; Nieuwenhuys and Oudejans, 2011b; Andersen et al., 2018). Research suggests that initial learning (e.g., skills acquisition) occurs best under low levels of stress (Driskell and Johnston, 1998; Driskell et al., 2008). However, skilled performance is typically learned through practice in settings that mimic the environment in which the skills will be performed operationally (Schmidt and Lee, 2013). For example, traditional firearms qualification scores have high congruency with other marksmanship assessments, but low congruency with the dynamic and rapidly unfolding nature of real-world OISs (Morrison and Vila, 1998; Wollert et al., 2011).

A well-established method for developing stress resilient skills and performance is stress exposure training (SET; Johnston and Cannon-Bowers, 1996; Driskell and Johnston, 1998; Driskell et al., 2008). SET is comprised of three carefully scaffolded phases: (1) information provision, (2) skills acquisition, and (3) application and practice, which encompass various techniques and components (Johnston and Cannon-Bowers, 1996; Driskell and Johnston, 1998; Driskell et al., 2008). The application and practice phase is typically achieved through scenario-based training (SBT), which provides officers a realistic, yet safe environment to make errors that, if made on-duty, could have severe consequences. SBT also allows officers to receive corrective feedback on their performance (Armstrong et al., 2014). The purpose of this phased approach is to increase knowledge of stress effects, reduce individuals’ anxiety and reactivity to stressors, and increase resources (e.g., skills schemas), confidence, and ability (e.g., coping) to perform under stress.

There is also growing evidence that decision-making accuracy and performance is not only related to increased sympathetic activity, but also the suppression of the stress modulating parasympathetic influence (Saus et al., 2006; Andersen et al., 2018). As such, police training that targets officers’ capacity to recognize and self-regulate their responses to stressors are demonstrating promise (McCraty and Atkinson, 2012). For example, Andersen et al. (2018) demonstrated that a physiologically focused intervention that taught police officers how to modulate SNS and PNS activation during SBT with real-time cardiovascular biofeedback led to significant reductions in lethal force decision-making errors and quicker physiological recovery from stress; improvements which were maintained over the 18 month study period.

While these training methodologies provide evidence of improved performance and increased resilience to the sorts of stress reactions observed in the current study, their adoption in policing is rare; in fact, stress-based training of any type appears to be used infrequently and training is seldom evidence-based or evaluated for intended outcomes (Sherman, 2015). For example, the authors are not aware of any studies that evaluate standard in-service police training for the alignment with the principles of SET. Furthermore, we could locate only one study that objectively measured levels of stress in training (Armstrong et al., 2014).

Armstrong et al.’s (2014) study examined four scenarios that were part of an agency’s mandatory UoF SBT. The results showed that, on average, HRs rose from 97 bpm pre-scenario to 116 bpm during physical contact. In contrast, the results of the current study found average HR between 116 and 142 bpm during the encounter/UoF/arrest phase (dependent on the incident factors present). This discrepancy highlights the value of research, like the sort presented in this paper. Our study indicates that the SBT training evaluated by Armstrong and colleagues may not be achieving its intended level of realism (i.e., training or testing skills under realistic conditions). The results of studies like ours can help inform the development and delivery of realistic and effective operational skills training that approximates real-world stress exposure. This evidence-based training approach is likely to be particularly important for improving performance in UoF encounters, which while low frequency (Hall and Votova, 2013; Baldwin et al., 2018), can result in tragic consequences and present substantial liability for officers and agencies (Braidwood, 2010; MacNeil, 2015; Dubé, 2016). It is also important to point out that studies like ours can also inform the development of SBT content, by informing agencies as to what sort of CFS and incident factors are occurring within their jurisdiction (e.g., if weapons are often accessible in CFS, that should be an element that is built into SBT scenarios).

### Arousal and Health

How occupational stress arousal impacts long-term health is also an area of avid interest and requires further investigation. Longitudinal research studies conducted with frontline officers have demonstrated elevated risks of chronic disease such as cancer, diabetes, and heart disease compared to populations of similar age (Violanti, 1983; Violanti et al., 2006b; Charles et al., 2007). Results described in this paper highlight the sorts of risks that officers are routinely exposed to in the course of their duties, while also revealing the nature of the stress reactions (and the frequency of these reactions) that may be at the root of some of these health concerns.

That being said, it is important to note that physiological arousal associated with high risk encounters (including those that involve UoF) may not necessarily be detrimental. In fact, it may be the case that higher levels of physiological arousal are appropriate (even preferred) in some encounters in order to meet the demands of the situation. What will be critical from a health risk standpoint, is not necessarily the level of arousal one experiences during the event, but *quick recovery* from the arousal (e.g., recovery within or shortly after the event). The frequency of high risk encounters in an officer’s shift, which was routinely observed in our study, may be problematic if it means that officers do not have time to recover fully during their active duty days. If this occurs, officers may experience accumulated stress that results in allostatic load, or “wear and tear” on the cardiovascular system, that is associated with long-term health outcomes (McEwen, 1998; Violanti et al., 2006b). Longitudinal research with police officers indicates that occupational stress is associated with chronic health outcomes such as cardiovascular and metabolic disease (Violanti et al., 2006b), but the study design does not allow for the distinction between the contribution of acute versus chronic stress to disease. Unfortunately, in the current study, we were unable to examine recovery rates and levels due to the varying and confounding nature of post-CFS activities (e.g., sitting, standing, reporting writing, immediately responding to another CFS) and inconsistent documentation of activities between calls (e.g., breaks, meals, interactions with officers/public). Thus, we cannot speak to health outcomes directly. Future research should certainly prioritize this so we can understand the long-term health implications of the “physiological profiles” that were generated from our study.

### Limitations

While we are optimistic about the use of these research findings to improve police training and health research, we caution future researchers and lay persons to interpret and use the findings with consideration given to study limitations. For example, there is significant public interest in understanding (and being able to explain) all police actions, particularly lethal encounters. Thus, there may be a temptation to use physiological arousal, as measured in research studies such as this, to find an individual officer culpable for their actions (e.g., “that officer was likely so stressed that their performance must have been compromised”). However, it is not appropriate to do this using research of the sort reported on here. For example, it is incredibly important to remember that *group level analyses* of stress, training, and performance can only be used to understand general relationships between these variables. While this understanding may be useful to improve police training, real-world performance, or overall health, in a general way, at no time are group level research findings on physiological arousal able to be used to explain why *one particular officer* acted in the way he/she did in the field.

While HR is the most easily monitored physiological measure of stress, we must stress that this is not an absolute measure of an individual’s stress, nor does it unequivocally predict individual performance under stressful conditions (Meyerhoff et al., 2004; Brisinda et al., 2015; Arble et al., 2019). Research equipment used to measure ambulatory physiological arousal in police research is not as accurate as tests used for diagnostic medical purposes (i.e., hospital grade ECG testing for cardiovascular disease), and therefore this measure must be interpreted with caution. Furthermore, the collection of additional biological indicators of the stress response (e.g., HPA activity, blood markers) was not possible in this real-world study as it may have interfered with the officer’s ability to meet the challenges of the emergency situation at hand. As heart rate reactivity is only one aspect of the stress response system, future research should include as much bio sampling as is logistically and ethically possible.

Above all else, research of this type (regardless of which recording device or bio-physiological measure one uses) cannot account for all the factors that likely go into an individual officer’s continuous risk assessment of a situation (which will likely include an assessment of subject behavior, environmental features, tactical considerations, etc.) and in the moment decision-making during a real-world encounter. Therefore, the appropriateness of an individual officer’s behavior in any particular encounter must be judged based on the *reasonableness* and *necessity* of their actions, given the totality of the circumstances.

## Conclusion

Very limited research exists that objectively measures stress reactivity experienced by police officers during active duty. This study provides several contributions to the field and adds to the dearth of research in this area. Of note, this study establishes the feasibility of using GPS and detailed operational police records to map general duty police officers’ autonomic stress responses to the phase of a call and incident factors. The use of this innovative approach, advanced statistical methods (i.e., LMM for repeated measures), and the ability to differentiate between physical and psychological stress (by controlling for movement), provides robust estimates of (psychologically related) physiological arousal to CFS factors (e.g., call priority, use-of-force). The research findings provide evidence of the extent and frequency of stress arousal in police operations, which has important implications for general duty policing, police training, and health research.

## Data Availability Statement

The datasets for this manuscript are not publicly available because of privacy and ethical restrictions. Requests to access the datasets should be directed to SB (simonbaldwin@cmail.carleton.ca).

## Ethics Statement

All procedures were approved by the Carleton University’s Research Ethics Board (REB #17-106853) and the agency’s Research Review Board (RRB).

## Author Contributions

SB and CB conceptualized the study. SB, TS, and BJ conducted the data collection. SB performed the data analysis and interpretation with guidance from JA and under the supervision of CB, and drafted the manuscript. CB, JA, TS, and BJ provided critical revisions. All authors approved the final version of the manuscript for submission.

## Conflict of Interest

The authors declare that the research was conducted in the absence of any commercial or financial relationships that could be construed as a potential conflict of interest.
